# Recurrent Giant Malignant Phyllodes Tumor of the Breast

**DOI:** 10.1155/2021/2476691

**Published:** 2021-08-19

**Authors:** Erik Kudela, Karol Kajo, Erik Kozubik, Tomas Rokos, Terezia Pribulova, Jan Danko, Eva Kudelova, Igor Homola, Pavol Slavik, Barbora Macurova, Kamil Biringer

**Affiliations:** ^1^Department of Obstetrics & Gynecology, Jessenius Faculty of Medicine in Martin, Comenius University in Bratislava, Slovakia; ^2^Department of Pathology, St. Elisabeth Cancer Institute, 812 50 Bratislava, Slovakia; ^3^Clinic of Surgery and Transplant Center, Jessenius Faculty of Medicine in Martin, Comenius University in Bratislava, Slovakia; ^4^Department of Plastic Surgery, Jessenius Faculty of Medicine in Martin, Comenius University in Bratislava, Slovakia; ^5^Institute of Pathological Anatomy, Jessenius Faculty of Medicine in Martin, Comenius University in Bratislava, Slovakia; ^6^Clinic of Radiology, Jessenius Faculty of Medicine in Martin, Comenius University in Bratislava, Slovakia

## Abstract

Phyllodes tumors (PTs) are rare fibroepithelial neoplasms of the breast. They have a proliferating stromal component that can be graded as benign, borderline, and malignant. In addition, they are associated with an increased risk of local recurrence and distant metastasis. The authors hereby present a case report of a 34-year-old woman with recurrent malignant PT with an increasing aggressiveness. There were two recurrences of giant tumors that consumed the entire right breast, which developed over a three-year period. The final surgical treatment was a total extirpation of the tumor with subsequent plastic reconstruction using a cutaneous flap from the region of the latissimus dorsi muscle. The patient died three months after the last recurrence due to multiorgan failure.

## 1. Introduction

Phyllodes tumors (PTs) are biphasic fibroepithelial neoplasms that have an exaggerated and prominent intracanalicular growth pattern with leaf-like stromal fronds covered by benign epithelium and accompanied by stromal hypercellularity [1]. PTs are rare, accounting for approximately 0.5% of all primary breast tumors [1, 2]. They often present as rapidly growing, painless masses [3]. Furthermore, based on histological criteria, PTs are graded as benign, borderline, and malignant, which represent only 8–33% of all PTs [1, 2, 4, 5]. Moreover, PTs are associated with an increased potential for local recurrence and distant metastasis [4]. However, their prognosis is unpredictable and is dependent on a comprehensive assessment of clinical and morphological characteristics.

We hereby present a case report of a 34-year old woman with recurrent malignant PT that has an increasing aggressiveness. She underwent oncoplastic surgery.

## 2. Case Presentation

A 34-year-old woman was admitted to the University Hospital in 2016. She had a tumor with a 7 cm diameter localized in the medial quadrants of the right breast. Subsequent tumor extirpation and final histological results showed a malignant PT with a mitotic activity of 15 mitotic figures per 10 high-power field (mf/10 hpf) but without heterologous structures. The patient had a total score of 57 points in the PT evaluation according to the Singapore nomogram (AMOS criteria–stromal atypia, mitotic activity, overgrowth, and status of surgical margins) and had an estimated five-year recurrence-free survival of 53% [6]. The patient refused any subsequent surgical treatment despite the presence of a positive margin at the base of the primary tumor.

Approximately two years later, the patient consulted again for the surgical treatment of a huge tumor with a diameter of 20 cm that completely consumed the entire right breast. The tumor had no signs of ipsilateral axillary lymphadenopathy. Computed tomography (CT) of the thorax, sonography of the liver, and bone scan were all negative for staging. Total mastectomy was performed with partial resection of the pectoral muscle due to tumor infiltration. The final histological finding was recurrent PT with solid and cystic components and highly accelerated mitotic activity (40 mf/10 hpf). Despite the presence of a positive margin, the patient refused any subsequent treatment.

**Figure 1:**
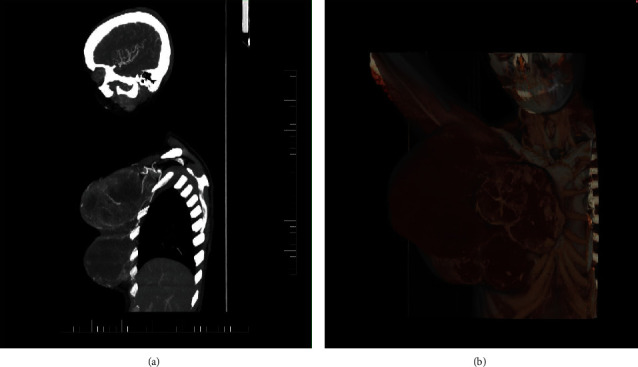
(a) Computer tomography (CT) arterial phase in the sagittal plane and (b) volume rendering technique with the fusion of venous phase. Partially seen magistral arteries and truncus costocervicalis feeding the tumor originating from the subclavian artery. The CT scan was performed during the last recurrence of the tumor.

**Figure 2:**
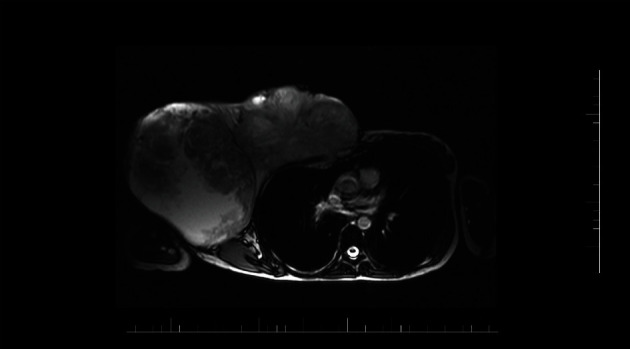
Magnetic resonance image (MRI T2 WI trufi tra) shows the overgrowth of the giant tumor into the thoracic wall at the level of the sternal ending of the third and fourth ribs with the thorax deformity. The MRI scan corresponds with the last recurrence of the tumor.

**Figure 3:**
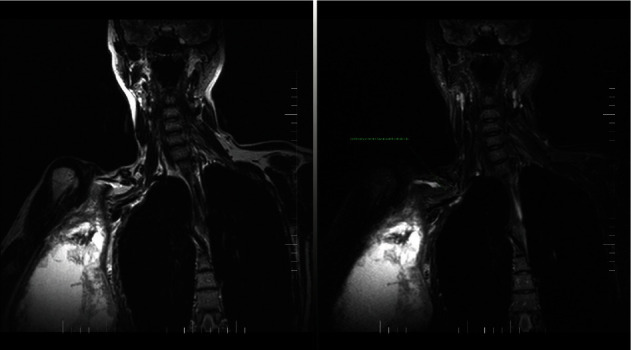
Magnetic resonance image (MRI T2 WI tse cor, STIR cor) shows the brachial plexus and its branches with a pathological signal. The MRI scan corresponds with the last recurrence of the tumor.

**Figure 4:**
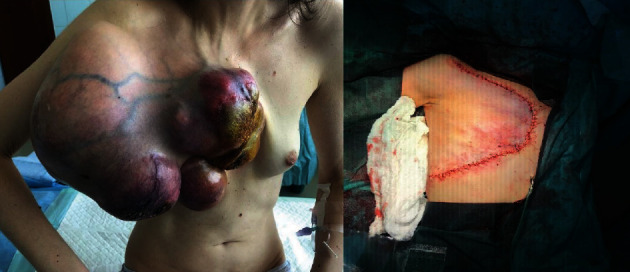
Giant tumor (second recurrence) affecting the right breast before and after the plastic reconstruction technique.

**Figure 5:**
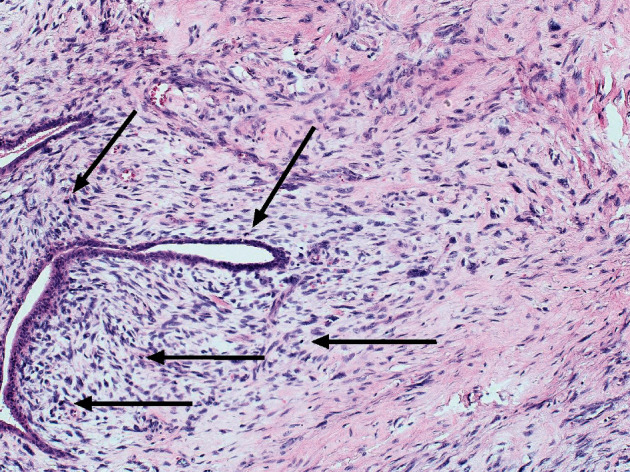
Microscopic character of phylloides tumor (PT) with increased cellularity around the glandular structures with signs of cellular atypia and increased mitotic activity (arrow-marked mitotic figures) (hematoxylin and eosin; magnification 100x).

**Figure 6:**
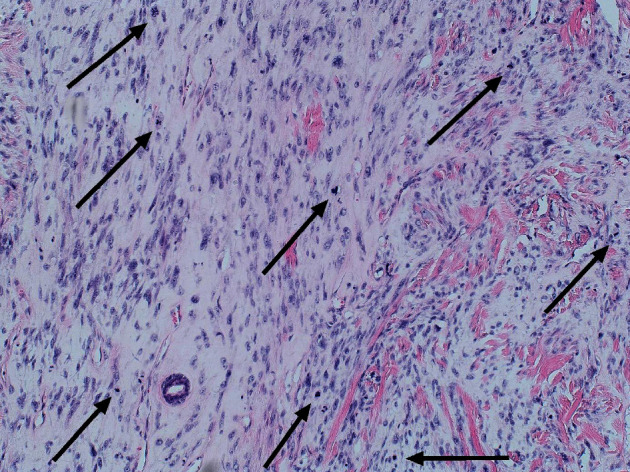
Histological image of malignant PT with accentuated mitotic activity. The arrows indicate the mitotic figures (hematoxylin and eosin; magnification 100x).

Finally, the patient returned 14 months after the second surgery with an exulcerated gigantic tumor of the right breast that grew towards the axilla. The CT and magnetic resonance imaging (MRI) scans of the patient showed that the tumor substantially compressed the thorax and had necrotic areas and gas bubbles. In addition, the tumor infiltrated the thorax at the sternal edge of the third and fourth ribs (Figures 1–3). The final surgical treatment conferred a high risk of damage to the venous and lymphatic drainage systems of the upper extremity with severe lymphedema. We performed a total extirpation of the tumor with subsequent plastic reconstruction using a cutaneous flap from the region of the latissimus dorsi muscle (Figure 4). The axillary nerves and vessels remained intact. A total of three separate tumor masses (20 × 15 × 15 cm, 19 × 11 × 10 cm, and 19 × 12 × 10 cm) were resected, and significant sarcomatous overgrowth and mitotic activity exceeding 50 mf/10 hpf were histomorphologically revealed (Figures 5 and 6). The base of the thorax was completely infiltrated by the tumor remnants that could not be removed. The patient refused any subsequent treatment and died a few months later due to multiorgan failure.

## 3. Discussion

PTs are unique mixed tumors that are characterized by clinical and morphological peculiarities. They are defined by considerable biological variability both in terms of their behavior and the spectrum of differentiation lines. PTs occur predominantly in older women with an average age of 40-50 years, which is about 15-20 years later than the average for fibroadenomas, which are in the same histogenetic group with PTs [1]. However, they may rarely occur in younger patients, as confirmed in our case.

Clinically, PTs usually present as painless, fast-growing masses. They should be suspected if the images from mammography and ultrasonography show a well-circumscribed oval or lobulated mass with rounded borders [7]. The median size of PTs is approximately 4 cm. However, less than 10% of tumors grow larger than 10 cm, and these have been defined as giant PTs [8, 9]. These are found in 20% of PTs [8]. Islam et al. [10] reported one of the largest giant PTs in the English literature, which was 50 × 50 cm in size. If we sum up the tumor sizes of the last recurrence affecting the breast described in our case report, it is an even larger entity than that reported by Islam et al. Large tumors distort the breast and may ulcerate the skin [10]. This was the case in the patient we hereby reported.

Once the diagnosis of PT is made, grading into benign, borderline, and malignant categories is accomplished based on assessing the constellation of histological criteria—degree of stromal hypercellularity, stromal atypia, mitotic activity, stromal overgrowth, and the nature of the tumor borders. The grade is important because of its correlation with the tumor's clinical behavior, particularly with local recurrences and metastases [4]. The local recurrence rates are 10–17%, 14–25%, and 23%, respectively, for benign, borderline, and malignant tumors, respectively, thereby indicating an increased risk of recurrence with increasing tumor grades [4]. In fact, up to 43.7% of originally benign tumors recur at higher grades [4]. Furthermore, metastases are reported to occur in 0.1%, 1.6%, and 16.7-22% of benign, borderline, and malignant PTs, respectively [1, 4, 11]. In addition, distant metastases usually develop within five to eight years after the diagnosis of PT. They have been reported in nearly all internal organs. However, the lungs and bones are the most common sites of spread [1]. In the case of our patient in this report, no distant metastases were observed on CT and MRI scans except for the local infiltration of the thoracic wall.

Intratumoral genetic heterogeneity is common in PTs and may account for the reported lack of correlation between histological grading and clinical behavior. The entire spectrum of molecular aberrations in PTs is yet to be fully defined. Tumor recurrence and progression are likely to reflect the presence of underrecognized subclones. p^16INK4a^ (*CDKN2A*) inactivation appears to be important in the pathogenesis of PT [12].

In most cases, the clinical course could not be predicted. Nevertheless, several tools have been created to express the risk of recurrence. The most famous of which is the Singapore nomogram, which is based on evaluating the so-called AMOS criteria (stromal atypia, mitotic activity, overgrowth, and status of surgical margins) [4, 6]. By evaluating these parameters, it is possible to assess the individual's risk. In our patient, a score of 57 points with a 53% recurrence-free survival was determined at the first tumor, which indicated an increased risk of recurrence.

The main differential diagnoses for malignant PTs are spindle cell metaplastic breast carcinomas and primary breast sarcomas [1, 4]. The distinction of malignant PT from metaplastic (sarcomatoid) carcinoma is based on the morphology and immunohistochemical detection of cytokeratins in carcinomatous cells [11]. Primary breast sarcoma is extremely rare, and most cases arise as a component of malignant PT [11]. However, the clinical outcomes of primary breast sarcomas and malignant PTs are similar [1].

The aggressive nature of malignant PT of the breast represents a therapeutic dilemma [4, 13]. The mainstay of PT treatment is complete surgical excision with negative margins. A meta-analysis reported that positive surgical margins were associated with local recurrence of malignant PTs. An overall local recurrence rate of 20.4% was documented for borderline and malignant PTs that were subjected to breast-conserving surgery with negative margins. This data was obtained from various studies that were combined [4].

A negative margin is an acceptable treatment following lumpectomy for PT. The National Comprehensive Cancer Network (NCCN) guidelines have advocated for wide local excision with margins of 1 cm or more without axillary staging to treat PT [14]. However, some practitioners consider this approach to be an overtreatment. An alternative would be the treatment of a positive margin with margin reexcision or close surveillance if they have a benign or borderline histology. Moreover, patients with a positive margin and malignant histology should undergo further surgery to obtain clear margins [15]. Our patient, despite having positive margins, did not agree to further reexcision; therefore, recurrence was highly probable.

Large skin defects require plastic reconstruction, including either a skin graft or rotation flap. However, the role of adjuvant therapy has not yet been established. While malignant and borderline PTs may undergo radiotherapy, administration of systemic chemotherapy is considered in individual cases [4]. The resistance of PTs to chemotherapy and radiotherapy remains unclear. Most patients with metastasis do not respond to standard chemotherapy and die within three years of initial treatment [11]. In these cases, alternative techniques include chemoembolization, resulting in successful tumor shrinkage [16].

PTs are rare mixed breast tumors. Their clinical course depends mainly on histomorphological characteristics and the adequacy of surgery. Therefore, these tumors require a very individual multidisciplinary diagnostic therapeutic approach and cooperation from the patient.

## Data Availability

Not applicable as this is a case report.
